# Association between ultra-processed foods consumption and micronutrient intake and diet quality in Iranian adults: a multicentric study

**DOI:** 10.1017/S1368980022002038

**Published:** 2022-10-24

**Authors:** Fahimeh Haghighatdoost, Parisa Hajihashemi, Noushin Mohammadifard, Farid Najafi, Hossein Farshidi, Masoud Lotfizadeh, Tooba Kazemi, Simin Karimi, Shahin Shirani, Kamal Solati, Nizal Sarrafzadegan

**Affiliations:** 1 Interventional Cardiology Research Center, Cardiovascular Research Institute, Isfahan University of Medical Sciences, Isfahan, Iran; 2 Isfahan Gastroenterology and Hepatology Research Center, Isfahan University of Medical Sciences, Isfahan, Iran; 3 Isfahan Cardiovascular Research Center, Cardiovascular Research Institute, Isfahan University of Medical Sciences, P. O. Box 81745-15, Isfahan, Iran; 4 Research Center for Environmental Determinants of Health, Health Institute, Kermanshah University of Medical Sciences, Kermanshah, Iran; 5 Hormozgan Cardiovascular Research Center, Hormozgan University of Medical Sciences, Bandarabbas, Iran; 6 Social Determinants of Health Research Center, Shahrekord University of Medical Sciences, Shahrekord, Iran; 7 Cardiovascular Diseases Research Center, Birjand University of Medical Sciences, Birjand, Iran; 8 Heart Failure Research Center, Cardiovascular Research Institute, Isfahan University of Medical Sciences, Isfahan, Iran; 9 Hypertension Research Center, Cardiovascular Research Institute, Isfahan University of Medical Sciences, Isfahan, Iran; 10 Department of Psychiatry, School of Medicine, Shahrekord University of Medical Sciences, Shahrekord, Iran; 11 Faculty of Medicine, School of Population and Public Health, University of British Columbia, Vancouver, Canada

**Keywords:** Ultra-processed food, Micronutrient, Nutrient adequacy, Diet quality, Hybrid nutrient density

## Abstract

**Objective:**

To identify ultra-processed foods (UPF) contribution to daily energy and nutrient intake in Iranians and examine whether UPF intake is associated with nutrient profile and diet quality.

**Design:**

In this cross-sectional study, a validated FFQ was used to evaluate usual dietary intake over the preceding year. NOVA system was applied to categorise foods based on their levels of processing. Diet quality was evaluated using the nutrient adequacy ratio (NAR), Nutrient Rich Food Index (NRF) and hybrid nutrient density.

**Setting:**

The LIPOKAP study conducted in five cities of Iran (Isfahan, Birjand, Bandar Abbas, Kermanshah and Shahrekord).

**Participants:**

A total of 1994 adults aged ≥18 years were recruited using stratified multistage random cluster sampling method.

**Results:**

UPF were responsible for 8·5 % of daily energy intake. In the adjusted model, UPF consumption was inversely associated with carbohydrate, protein, refined and whole grains, fibre, fruit and meat, but was positively linked to energy, total fat, saturated and trans fatty acids and cholesterol. Compared with those in the lowest tertile, individuals in the highest tertile of UPF had smaller NAR for Ca, Mg, Zn, Fe, phosphorus, thiamin, niacin, folate and vitamin C. Both NRF and hybrid nutrient density decreased when the share of daily energy intake from UPF increased.

**Conclusion:**

The higher consumption of UPF is associated with poorer diet quality and lower nutrient intake. It is recommended that UPF be replaced with minimally processed foods to improve diet quality and nutrient profile.

Ultra-processed foods (UPF) are produced through some industrial techniques or chemical synthesis. They derive from whole foods and contain large amounts of ingredients and refined foods to enhance the sensory properties of foods^(1)^. Nevertheless, changes in the food processing over the last years, their wide availability and their low-dependence on culinary preparation have led to a considerable rise in their popularity and consumption^(2)^. This, in turn, has caused adverse impacts on food systems, nutrients intake and health status^(2)^. UPF mainly contain high amounts of fat, added sugar and salt^(3)^. Mounting evidence suggests a positive link between UPF and mortality^(4)^, CVD^(5)^, metabolic syndrome, depression and cancer^(6)^. Therefore, current dietary guidelines have emphasised eliminating UPF and reducing processed foods (PF) consumption^(7,8)^.

Despite some debates about categorising foods based on the extent to which they are processed^(9)^, the NOVA system is widely used in epidemiological studies. Accordingly, foods are categorised in one of the following categories: (1) unprocessed or minimally processed foods (MPF); (2) processed culinary ingredients (PCI); (3) PF; and (4) UPF and drink products^(10)^. The contribution of UPF to daily energy intake varies from 10 % in Portugal^(11)^ to around 60 % in the USA^(12)^ and in the UK^(13)^. It is of note that poor nutrient profile of UPF^(13–16)^ is a main concern of UPF consumption beside the adverse effects of altered food structure, additives and neo-formed contaminants caused by processing^(5)^. Conversely, decreasing UPF consumption^(17)^ and replacing them with MPF^(16)^ improved diet quality in Americans and Canadians, respectively. Despite these studies, there is evidence indicating that some of the UPF are required to meet nutrient adequacy^(18)^. In a population-based study in the Washington state, UPF were the main source of plant protein and vitamin E, thiamin, niacin, folate and Ca^(18)^. In addition, PF had considerable share to both micro- and macronutrient intake^(9,18)^, and minimising their consumption was associated with nutrient deficiency^(9)^.

Although many investigations have studied dietary share of all NOVA categories in daily energy and nutrient intake in different populations, there is a lack of information in this regard among Iranians. This is of note since UPF consumption is determined not only by the socioeconomic status but also by the variety and availability of them. Therefore, examining such association in a low-income country, where UPF are less diverse compared with developed countries, would provide new insight into the nutrition policy actions in low-income countries.

To fill this gap, the present study aims to identify the contribution of all NOVA categories to daily energy and nutrient intake in a multicentric study among Iranians and examine whether the higher share of UPF in daily energy intake is associated with nutrient profile and diet quality.

## Methods

### Study population

The knowledge and practice of dyslipidaemia prevention, management and control (LipoKAP) project is a community-based trial conducted from February 2018 to July 2019^(19)^. In the present analysis, we used its baseline data as a cross-sectional study. Within the LipoKAP project, a total of 2456 adults aged ≥18 years were recruited from five different cities of Iran (Isfahan, Birjand, Bandar Abbas, Kermanshah and Shahrekord). The participants were selected using stratified multistage random cluster sampling method. Accordingly, the adequate sample size was estimated through the simple random method and then doubled because of having different clusters. Considering the distribution of population in different cities and in the urban and rural areas of each city, the final sample size for each area was determined. Then, clusters were randomly selected from among available clusters in health care centres. Based on the distribution of population across different clusters, a specific sample size for each cluster was allocated, and participants were randomly selected and invited by the interviewers. All interviewers had already participated in a 4-h educational session. All apparently healthy adults (≥ 18 years) were eligible to be recruited in our study. Those with any systemic or dyslipidaemia-related diseases (such as fatty liver, peripheral vascular diseases and CVD (myocardial infarction or stroke)), chronic kidney disease, liver disease, cancer, immune system disorders and under- or over-estimation of energy intake (< 800 or > 4200 kcal/d) were excluded (*n* 462). These exclusion criteria were applied to mitigate the reverse causality caused by their potential confounding effect. All participants provided a written informed consent before participating in this study. This study was approved by the ethic committee of Isfahan University of Medical Sciences (registration number: IR.MUI.RC.1395.4.077). A self-administered questionnaire was used to assess socio-demographic information including age, gender, smoking and socio-economic status. More details regarding study participants and design have been provided elsewhere^(19)^. Physical activity was estimated using the International Physical Activity Questionnaire^(20)^. According to the median of physical activity level (1680 metabolic equivalent h/week), participants were categorised as physically active and inactive. Participants were directly asked to report on average, how many h/d they usually sleep. Sleep duration less than 8 h/d was regarded as short sleep duration^(21)^. A self-administered instrument EQ-5D was used to assess the contributors’ quality of life (QOL). The EQ-5D contains five domains of health status: mobility, self-care, usual activities, pain/discomfort and anxiety/depression. Three distinct levels of severity presented for each domain as: 1 (No problems); 2 (some problems) and 3 (extreme problems). Higher EQ-5D scores indicate poor QOL^(22)^.

### Dietary assessment

A validated, semi-quantitative FFQ was used for assessing habitual dietary intake of participants over the preceding year^(23)^. A popular portion size, derived from earlier Persian FFQ, was considered for each foodstuff, and participants were asked to determine how often they consumed each foodstuff last year. Participants could choose the best match answer amongst nine possible categories, from never/seldom to more than 6 times/d. The average intake of each food item (g/d) was computed for all participants according to the weight of each portion size and the frequency of consumption. Then, daily intake of energy and nutrients was calculated by means of Nutritionist IV software (version 7.0; N-Squared Computing) which was adjusted for Iranian foods^(24)^.

According to the NOVA classification system^(13,14)^, each foodstuff was classified into one of the following four groups: MPF (e.g. fruits, vegetables, milk and yogurt, meat, egg, legumes and beans, pasta and unsalted nuts and seeds), PCI (e.g. butter, oils, lard, salt and sugar), PF, which are obtained by combining foods of the first two groups (e.g. cheese, dough, curd, pickles, canned vegetables and beans, canned fish, bread and salted nuts and seeds) and UPF, which are formulated using several ingredients, additives and a series of industrial processes (e.g. pizza cheese, margarine, ice-cream, tomato paste, spreads and sauces, bologna sausage, biscuits, chips, cake, confectionary, chocolate, dessert, sugar sweetened drink and artificial juice drink). Since ready-to-heat pasta is not popular in Iran, and it needs to be prepared at home, it was categorised into the category of MPF.

### Nutrient adequacy ratio

The nutrient adequacy ratio (NAR) was calculated by dividing daily intake of each nutrient by the standard recommended amounts^(25)^ for their age and sex. The NAR was estimated for fourteen minerals (potassium, Ca, Mg, Zn, Fe and phosphorus) and vitamins (thiamin, riboflavin, niacin, pyridoxine, folate, cobalamin and vitamins C and D).

### Nutrient rich food index 6·3 (NRF 6·3)

The NRF 6·3 was computed by using the following formula^(26)^:

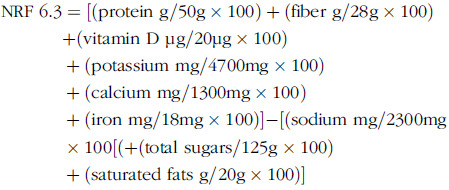




### Hybrid nutrient density score estimation

Hybrid Nutrient Density Score, as a diet quality measure, which considers both nutrients and food groups simultaneously, was calculated by using the following formula^(26)^:

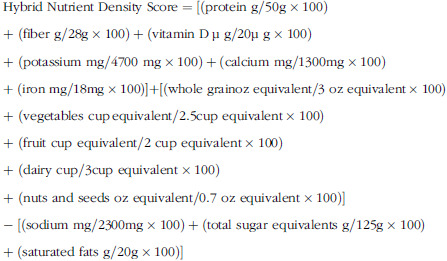




### Statistical analysis

Participants were categorised based on different variables of socio-demographic variables. The percentage contributions of all NOVA groups to daily energy intake of participants between different subgroups were compared using independent sample *t*-test or one-way ANOVA.

The nutritional content of the diet and diet quality scores across the tertiles of UPFcontribution to daily energy intake were compared by ANOVA in crude and ANCOVA in multivariable-adjusted models. The model was adjusted for age (continuous), sex and energy intake (continuous). Nutrient adequacy ratios were adjusted for further variables including education, physical activity, smoking, sleep duration and quality of life. Data were expressed as mean and sd or se where applicable. The mean percentage contributions of NOVA categories to daily nutrients intake were estimated using descriptive tests. All analyses were performed using SPSS Statistics (Version 23.0. IBM Corp.), and statistical significance was set at *P* value < 0·05.

## Results

Overall, 1994 subjects (female = 55 %) were included in the final analysis. The proportion of different NOVA-based food categories in total daily energy intake across participants’ socio-demographic characteristics is displayed in Table 1. Overall, MPF and PF were the first and second contributors of daily energy intake with a figure of almost 40 % and 34 %, respectively. MPF accounted for about half of the usual daily energy intake in both sexes, although the percentage of total energy intake in females was slightly higher than that of in males. Usual energy intake from PF was higher in males compared with females. Consumption of PCI and PF increased with age. In contrast, usual energy intake from UPF decreased in older participants. MPF and UPF consumption was higher in participants with higher levels of education. Conversely, a reduction in PCI and PF consumption was observed by education levels. Usual energy intake from MPF and PF decreased as the sleep duration increased. In contrast, PCI and UPF consumption was higher in participants with longer sleep duration. In terms of QOL, only UPF consumption was significantly higher in participants with higher QOL, and no difference was observed for other NOVA categories. MPF were consumed in higher amounts by non-smokers, while their PF and UPF consumption was lower than that of in current smokers. Physically inactive participants had higher intake of UPF and PCIs, but lower PF consumption than physically active participants.

**Table 1:** Contribution of different NOVA-based food categories in total daily energy intake according to socio-economic variables strata

	*n*	MPF (% total energy intake)		PCI (% total energy intake)		PF (% total energy intake)		UPF (% total energy intake)	
		Mean	sd	Mean	sd	Mean	sd	Mean	sd
All	1994	39·88	10·54	15·15	9·31	34·16	10·98	8·44	6·00
Sex									
Men	979	38·69	10·18	14·74	9·05	35·65	10·60	8·57	5·82
Women	1064	40·87	10·72	15·44	9·59	32·78	11·13	8·51	6·43
*P* value*		<0·0001		0·090		<0·0001		0·835	
Age									
<40 years	1124	39·63	10·20	14·58	8·46	33·37	10·68	9·93	6·17
≥40 years	894	39·97	10·90	15·85	10·36	35·18	11·27	6·75	5·55
*P* value*		0·473		0·003		<0·0001		<0·0001	
Education									
≤5 years	455	36·90	10·78	19·46	12·24	35·17	11·50	6·21	5·92
6–12 years	939	40·03	10·40	14·28	8·28	34·69	11·02	8·66	5·88
≥13 years	649	41·57	10·07	13·26	7·24	32·65	10·37	10·00	6·19
*P* value†		<0·0001		<0·0001		<0·0001		<0·0001	
Sleep duration									
<8 h	604	42·22	10·21	12·47	7·89	35·32	10·38	7·74	5·73
≥8 h	1443	38·83	10·48	16·20	9·67	33·66	11·17	8·88	6·28
*P* value*		<0·0001		<0·0001		0·001		<0·0001	
Quality of life									
Low	732	40·06	10·95	15·09	10·08	34·67	11·31	7·85	6·42
High	1306	39·69	10·26	15·15	8·90	33·82	10·76	8·94	5·95
*P*-value*		0·452		0·902		0·093		<0·0001	
Current smoker									
Yes	228	36·31	9·85	15·32	9·55	36·82	10·19	9·31	6·04
No	1766	40·34	10·54	15·13	9·29	33·82	11·04	8·33	6·00
*P* value*		<0·0001		0·777		<0·0001		0·022	
Physical activity									
Active	996	39·78	10·25	14·37	7·98	35·52	10·45	8·05	5·80
Inactive	998	39·99	10·83	15·92	10·43	32·81	11·34	8·84	6·18
*P* value*		0·657		<0·0001		<0·0001		0·003	

MPF: unprocessed or minimally processed foods; PCI: processed culinary ingredients; PF: processed foods; UPF: ultra-processed foods.

* Derived from independent sample *t*-test.

† Derived from ANOVA.

Data are expressed as mean ± sd.

Dietary intakes of participants across the tertiles of UPF are summarised in Table 2. Higher tertiles of UPF were significantly associated with lower usual consumption of MPF and PF. Conversely, the usual intake of UPF increased across the tertiles. Energy, total fat, SFA, PUFA, MUFA, trans fat and cholesterol intakes were higher in the top tertile of UPF, while those in the lowest tertile had higher intake of carbohydrate, protein and fibre. Whole grains, refined grains, fruits and meat were consumed in fewer amounts by individuals in the highest tertile of UPF. Vegetables intake tended to be lower in the highest tertile of UPF compared with the lowest tertile (318·01 ± 4·97 *v*. 301·24 ± 4·96 g/d; *P* = 0·052). Hybrid nutrient density, usual intake of potassium, Ca, Mg, Zn, Fe, phosphorous, Na, thiamin, riboflavin, niacin, folate and vitamin C decreased across the tertiles of UPF, whereas cobalamin intake went up. The percentage of participants receiving less than dietary recommended allowance for each nutrient across the tertiles of UPF has been shown in online Supplemental Table 1. Except for thiamin, the trend for all nutrients was downward.

**Table 2:** Food groups and nutrients across the tertiles of UPF contribution to total energy intake*

	UPF (% TEI) tertiles						*P* value†
	T1		T2		T3		
	Mean	se	Mean	se	Mean	se	
Energy (kcal/d)	2114·48	27·32	2304·33	26·46‡	2372·17	27·38‡	<0·0001
MPF (% TEI)	41·22	0·42	40·51	0·40	37·60	0·41‡,§	<0·0001
PCI (% TEI)	15·41	0·37	14·73	0·35	15·31	0·36	0·339
PF (% TEI)	38·51	0·40	34·97	0·39‡	29·00	0·40‡,§	<0·0001
UPF (% TEI)	2·82	0·12	7·39	0·12‡	15·40	0·12‡,§	<0·0001
Carbohydrate (g/d)	290·54	1·72	281·82	1·65‡	274·66	1·70‡,§	<0·0001
Total fat (g/d)	87·96	0·83	91·31	0·80‡	96·42	0·83‡,§	<0·0001
Saturated fat (g/d)	30·94	0·47	33·07	0·45‡	36·17	0·47‡,§	<0·0001
Poly unsaturated fat (g/d)	25·54	0·37	25·95	0·36	27·44	0·37‡,§	0·001
Mono unsaturated fat (g/d)	26·48	0·31	27·96	0·30‡	29·69	0·31‡,§	<0·0001
Trans fat (g/d)	2·42	0·27	2·80	0·26	3·95	0·27‡,§	<0·0001
Cholesterol (g/d)	264·71	4·32	288·84	4·15‡	302·39	4·27‡	<0·0001
Protein (g/d)	97·19	0·67	96·51	0·64	89·68	0·66‡,§	<0·0001
Fibre (g/d)	23·62	0·19	23·23	0·19	20·90	0·19‡,§	<0·0001
Hybrid nutrient density	674·89	11·68	656·81	11·24	565·05	11·67‡,§	<0·0001
Food groups							
Whole grains (g/d)	108·71	3·81	93·53	3·66‡	66·44	3·77‡,§	<0·0001
Refined grains (g/d)	259·39	4·87	236·49	4·68‡	205·44	4·82‡,§	<0·0001
Dairy products (g/d)	364·58	8·47	351·10	8·13	338·08	8·38	0·100
Vegetables (g/d)	318·01	4·97	313·80	4·78	301·24	4·96	0·052
Fruit (g/d)	262·58	5·29	251·13	5·08	222·63	5·23‡,§	<0·0001
Legumes (g/d)	51·65	1·52	52·41	1·46	50·50	1·51	0·657
Nuts and seeds (g/d)	24·74	0·96	26·46	0·92	24·90	0·95	0·345
Fish, Sea food (g/d)	14·73	0·63	16·00	0·61	15·59	0·62	0·351
Meat (g/d)	75·98	1·68	76·06	1·61	67·49	1·66‡,§	<0·0001
Micronutrients							
Potassium (mg/d)	3505·10	27·52	3496·86	26·43	3376·28	27·22‡,§	0·001
Na (mg/d)	2143·73	21·35	2073·79	20·53	2022·75	21·32‡	0·001
Ca (mg/d)	1085·58	12·48	1080·84	11·98	1038·16	12·34‡,§	0·013
Mg (mg/d)	385·52	3·73	382·01	3·58	350·44	3·69‡,§	<0·0001
Zn (mg/d)	15·23	0·16	15·13	0·15	14·31	0·16‡,§	<0·0001
Fe (mg/d)	16·18	0·13	16·08	0·12	14·58	0·13‡,§	<0·0001
Phosphorous (mg/d)	1588·58	12·15	1581·04	11·66	1506·92	12·01‡,§	<0·0001
Thiamin (mg/d)	1·60	0·01	1·58	0·01	1·39	0·01‡,§	<0·0001
Riboflavin (mg/d)	1·66	0·02	1·68	0·02	1·61	0·02§	0·016
Niacin (mg/d)	19·36	0·17	18·82	0·16	17·12	0·17‡,§	<0·0001
Pyridoxine (mg/d)	4·39	0·15	4·16	0·14	3·91	0·15	0·080
Folate (µg/d)	295·34	2·37	294·42	2·28	266·11	2·35‡,§	<0·0001
Cobalamin (µg/d)	3·79	0·09	4·19	0·09‡	3·89	0·09	0·005
Vitamin C (mg/d)	125·23	2·00	128·26	1·91	117·12	1·97‡,§	<0·0001
Vitamin D (µg/d)	2·18	0·07	2·28	0·06	2·21	0·06	0·481

* The nutrients and food groups were adjusted for age, sex and total energy intake (kcal). Energy intake was adjusted for age and sex. Data are expressed as mean ± se. UPF (% TEI), contribution to percentage of total daily energy intake from ultra-processed foods.

† Derived from ANCOVA.

‡ Significant difference with tertile 1.

§ Significant difference with tertile 2.

**Fig. 1:**
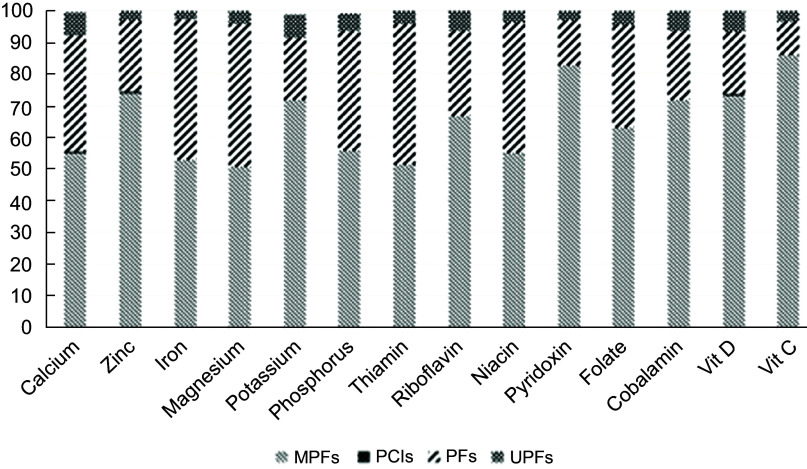
The contribution of all NOVA categories in daily intake of different nutrients

The contribution of all NOVA categories in daily intake of different nutrients is shown in Fig. 1. PCI were responsible for just a small fraction of all nutrients intake (less than one percent). UPFcame second and contributed to almost three percent (vitamin C and Zn) to around seven percent (Ca and potassium) of daily nutrients intake. The contribution of PF in daily nutrient intake varied between 11 % (vitamin C) to just over 40 % (thiamin, niacin, Mg and Fe). MPF had the greatest role in daily intakes for all nutrients which ranged from around a half of daily intake (thiamin, niacin, Ca, Mg, Fe and phosphorus) to above four-fifths (vitamin C and pyridoxine).

Table 3 provides means and se for NAR of nutrients in crude and multivariate-adjusted model across tertiles of UPF. In the crude model, compared with individuals in the bottom tertile, those in the top tertile of UPF had higher NAR for Ca (1·06 ± 0·02 *v*. 0·93 ± 0·02; *P* < 0·0001), Mg (1·04 ± 0·02 *v*. 0·97 ± 0·02; *P* < 0·0001), Zn (1·65 ± 0·03 *v*. 1·48 ± 0·00; *P* < 0·0001), phosphorous (2·26 ± 0·03 *v*. 2·06 ± 0·03; *P* < 0·0001), niacin (1·21 ± 0·02 *v*. 1·19 ± 0·02; *P* = 0·003), folate (0·69 ± 0·01 *v*. 0·68 ± 0·01; *P* < 0·0001) and vitamin C (1·45 ± 0·03 *v*. 1·40 ± 0·03; *P* = 0·0015). When lifestyle confounders were taken into account, the trend was reversed, and NAR tended to decrease across the tertiles of UPF. Furthermore, individuals in the top tertile of UPF had lower means of NAR for Fe (1·42 ± 0·02 *v*. 1·56 ± 0·02; *P* < 0·0001) and thiamin (1·20 ± 0·01 *v*. 1·38 ± 0·01; *P* < 0·0001) than those in the bottom tertile. The means of NAR for potassium (T3: 0·75 ± 0·01 *v*. T1: 0·69 ± 0·01; *P* < 0·0001), riboflavin (T3: 1·41 ± 0·02 *v*. T1: 21·27 ± 0·02; *P* < 0·0001) and vitamin D (T3: 0·16 ± 0·00 *v*. T1: 0·13 ± 0·00; *P* < 0·0001) were higher in individuals in the highest tertile of UPF compared with those in the lowest tertile. However, after adjustment for potential confounders, these associations remained no longer significant. Regarding cobalamin, those in the second tertile had the highest NAR, and means for the first and third tertile were not statistically different (T3: 1·60 ± 0·04, T2: 1·71 ± 0·04, T1: 1·55 ± 0·04; *P* = 0·009).

**Table 3:** Nutrient adequacy ratios for various nutrients across the tertiles of UPF contribution to total energy intake

	UPF (% TEI) tertiles						*P* trend*
	T1		T2		T3		
	Mean	se	Mean	se	Mean	se	
Potassium							
Crude	0·69	0·01	0·75	0·01	0·75	0·01	<0·0001
Adjusted model†	0·74	0·01	0·73	0·001	0·71	0·01	0·08
Ca							
Crude	0·93	0·02	1·06	0·02	1·06	0·02	<0·0001
Adjusted model	1·03	0·01	1·03	0·01	0·99	0·01	0·020
Mg							
Crude	0·97	0·02	1·06	0·02	1·04	0·02	<0·0001
Adjusted model	1·06	0·01	1·04	0·01	0·97	0·01	<0·0001
Zn							
Crude	1·48	0·00	1·64	0·02	1·65	0·03	<0·0001
Adjusted model	1·62	0·02	1·60	0·02	1·54	0·02	0·004
Iron							
Crude	1·46	0·03	1·62	0·04	1·45	0·03	0·001
Adjusted model	1·56	0·02	1·54	0·02	1·42	0·02	<0·0001
Phosphorous							
Crude	2·06	0·03	2·29	0·03	2·26	0·03	<0·0001
Adjusted model	2·24	0·02	2·22	0·02	2·14	0·02	<0·0001
Thiamin							
Crude	1·27	0·02	1·37	0·02	1·27	0·02	<0·0001
Adjusted model	1·38	0·01	1·33	0·01	1·20	0·01	<0·0001
Riboflavin							
Crude	1·27	0·02	1·42	0·02	1·41	0·02	<0·0001
Adjusted model	1·37	0·02	1·39	0·02	1·34	0·02	0·075
Niacin							
Crude	1·19	0·02	1·27	0·02	1·21	0·02	0·003
Adjusted model	1·29	0·01	1·24	0·01	1·14	0·01	<0·0001
Pyridoxine							
Crude	2·96	0·11	3·12	0·10	3·03	0·11	0·586
Adjusted model	3·10	0·11	3·06	0·10	2·94	0·11	0·585
Folate							
Crude	0·68	0·01	0·74	0·01	0·69	0·01	<0·0001
Adjusted model	0·73	0·01	0·73	0·01	0·66	0·01	<0·0001
Cobalamin							
Crude	1·38	0·04	1·77	0·05	1·72	0·04	<0·0001
Adjusted model	1·55	0·04	1·71	0·04	1·60	0·04	0·009
Vitamin C							
Crude	1·40	0·03	1·52	0·03	1·45	0·03	0·015
Adjusted model	1·47	0·02	1·49	0·02	1·40	0·02	0·016
Vitamin D							
Crude	0·13	0·00	0·15	0·001	0·16	0·00	<0·0001
Adjusted model	0·14	0·00	0·15	0·001	0·14	0·00	0·572

* Resulted from ANOVA in the crude model and ANCOVA in the adjusted model.

† Adjusted for age, sex, energy, education, physical activity, smoking, sleep duration and quality of life.

Data are expressed as mean ± se.

UPF (% TEI), contribution in percentage of total daily energy intake from ultra-processed foods.

Further subgroup analysis based on various covariates (listed in Table 1) showed that vitamin D, potassium and acid folic were not adequately met in any of the subgroups irrespective of the amount of UPF, while Ca and Mg differed by the subgroups. In the higher tertile of UPF, Ca was not adequately met in women, older adults, subjects with low educational level (<5 years), both short and long sleepers, physically active subjects, people with low QOL and non-smokers, whereas in the counterpart group, NAR for Ca with increasing UPF consumption was not a concern. Mg was also consumed less than the RDA in the top tertile of UPF in men, older adults, people with lower educational attainment (< 5 and 5–12 years), long sleepers, physically active subjects, people with both low and high QOL and both smokers and non-smokers. Except for niacin in low-educated people (<5 years), Fe in women and vitamin C in smokers which were not met with increasing UPF consumption, there was not any more concern regarding other nutrients in different subgroups (data not shown).

**Fig. 2:**
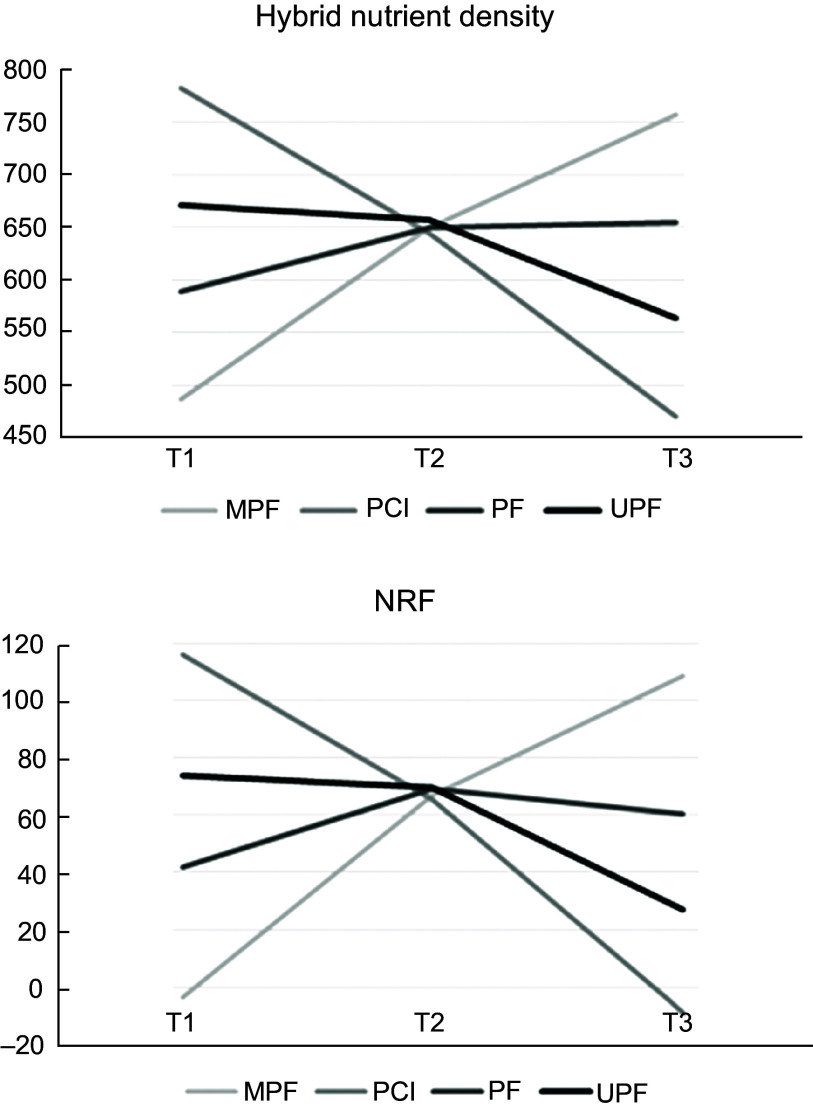
The Nutrient Rich Food Index (NRF) and hybrid nutrient density changes across the tertiles of all NOVA categories

NRF and hybrid nutrient density changes across the tertiles of all NOVA categories are revealed in Fig. 2. Overall, the trend of changes in NRF and hybrid nutrient density was almost identical for all NOVA categories. There was a constant downward trend for both NRF and hybrid nutrient density across the tertiles of UPF and PCI, while both continuously went up with increasing MPF intake. In terms of PF, despite a moderate rise in both indices in tertile 2 compared with the first tertile, moving on the third tertile led to a drop in both NRF and hybrid nutrient density.

## Discussion

This study was conducted to estimate the share of all NOVA categories in daily energy and nutrient intake in Iranians and explore how the quality of diet changes with increasing UPF intake. Average daily energy intake from UPF was around 8·5 %, and MPFs along with PFs contributed to over 70 % of daily intakes of energy and most nutrients. After adjustment for potential confounders, there was a linear inverse association between UPF and NAR for Fe, Zn, Ca, Mg, phosphorus, potassium, vitamins B_1_, B_3_, B_9_ and C. NRF and hybrid nutrient density, as a measure of overall diet quality, also decreased by increasing UPF consumption.

According to available evidence, Portugal, with an average intake of 10 %, has the lowest contribution of UPF to daily energy intake in the world^(11)^. In our study population, the average share of UPF was even less than Portugal. UPF intake is influenced by several factors, such as socio-economic status and culture and therefore differs from one population to another. Home cooking is common among Iranians like Portuguese, which can result in lower consumption of UPF. In addition, the effects of sanctions, recession and inflation, over the past 5 years, on all aspects of Iranians’ lives, in particular dietary intakes, cannot be neglected. The higher prices of UPF compared with MPF might be an explanation for their low intake in this population, as the earlier evidence has suggested an inverse association between the prices of UPF and their consumption^(27)^. In support of this, according to our earlier study conducted in 2013–2014, UPF were accounted for around 20 % of daily energy intake (Accepted article). In terms of other determinants of UPF intake, we found a positive association between educational level and UPF consumption and an inverse link between age and UPF consumption which were compatible with the results of other developed, high-income countries^(14,15,28)^.

This study also revealed that higher UPF consumption was accompanied by lower NAR for different nutrients, NRF and hybrid nutrient density score. This is principally attributable to lower intake of nutrient-dense foods, such as fruit, vegetables, whole grains and fibre. On the contrary, higher intake of fats and oils in the higher tertiles of UPF causes higher energy intake without improving dietary nutrient profile. These results are consistent with earlier findings in different populations^(14,15)^. In the I.Family study, both children and adults with higher UPF intake consumed higher amounts of sugar and fat but lower amounts of fibre and protein^(14)^. In Portuguese adults and the elderly, higher amounts of UPF consumption were associated with higher carbohydrates, saturated fats and sugar, while the intake of protein, fibre, vitamin A, vitamin C, folate, potassium, Mg and Fe was lower in comparison with a healthy traditional dietary pattern^(15)^. Furthermore, in our subgroup analysis, NAR of Ca and Mg were closely related to various lifestyle and socio-economic variables. On the other hand, the inverse association between UPF consumption and Ca and Mg intake differed between different categories of a specific variable. Although with increasing UPF intake, meeting the RDA for Fe in women, niacin in low-educated people and vitamin C in smokers were a matter of concern, they were adequately met in other categories irrespective of the amount of UPF.

Assessing hybrid nutrient density in this study provides an overview of both nutrients and food group’s intake. In other words, earlier diet quality indices such as the Mediterranean diet score and healthy eating score are merely based on food groups, while nutrient mean intake and nutrient density fail to provide any information regarding the consumption of food groups. Additionally, hybrid nutrient density score concurrently considers nutrients to limit and to encourage. Studies examining UPF in relation to either nutrient density or healthy eating index revealed an inverse association^(28–30)^, which support our findings. SFA intake was upper than the recommended limit in all three tertiles, while trans fatty acids consumption was around 1 to 1·5 percent of total daily energy intake. Although Na intake was lower than the tolerable upper intake level in all three categories of UPF, it should be taken into account that these values are only Na content of foods and our FFQ failed to examine the salt intake of participants. The inverse trend between UPF consumption and Na intake in this population, which is similar to the USA^(17)^, might be owing to sweet products which largely contribute to UPF intake.

This study has several strengths. First, this is the first report from a low-income, developing country where home cooking is still common. Given that public education interventions and food environment regulatory policies can potentially change the nutrient content of UPF, our findings are relevant. Second, this is a multicentric study from different cities, which increases the external validity of our findings. Third, we assessed overall diet quality using hybrid nutrient density that simultaneously emphasise on nutrients and food groups to encourage and nutrients to limit and explains over 70 % of variance in total HEI-2015^(31)^. Finally, the various socio-demographic variables were adjusted to minimise their confounding effects. The limitations of the present study include its cross-sectional design, estimating nutrient and energy intakes by means of the US Department of Agriculture’s food composition table, relying on self-reported data, using a memory-dependent questionnaire to assess dietary intake and failing to rule out the effect of unmeasured and unknown confounders. Moreover, it should be kept in mind that FFQ cannot provide precise estimation of Na intake, and we did not measure salt intake. On the other hand, the estimated Na intake is only based on foods’ Na content which cannot exactly show how much Na was consumed by participants.

In conclusion, this study shows the small proportion of UPF in Iranians’ daily energy intake. The higher consumption of UPF, the lower diet quality and nutrients density scores. Although PF’ contribution to nutrient intake was considerable, the main source of all nutrients was MPF. In addition, increasing the MPF consumption was associated with a constant rise in both NRF and hybrid nutrient density, while the figures remained fairly constant for PF after a medium increase. Therefore, it is strongly recommended that UPF be replaced with MPF to improve diet quality.

## Supplementary material

For supplementary material accompanying this paper visit https://doi.org/10.1017/S1368980022002038. click here to view supplementary material
