# Supplementary material for: Association between ultra-processed foods consumption and micronutrient intake and diet quality in Iranian adults: a multicentric study
Source: Public Health Nutr. 2022 Oct 24;26(2):467–75. doi: 10.1017/S1368980022002038 (PMC13076090; doi:10.1017/S1368980022002038)
Supplement: Supplementary file 1 [file S1368980022002038sup001.docx]

Supplementary table 1- The percentage of people receiving less than EAR for each nutrient.

|  | **UPFs tertiles** | | |  |
| --- | --- | --- | --- | --- |
|  | **T1** | **T2** | **T3** | **P trend^1^** |
| Potassium | 89.30 | 85.50 | 83.20 | 0.005 |
| Calcium | 62.30 | 48.60 | 47.20 | <0.0001 |
| Magnesium | 58.00 | 49.00 | 50.50 | 0.002 |
| Zinc | 23.90 | 15.90 | 17.50 | <0.0001 |
| Iron | 35.90 | 35.20 | 39.40 | 0.245 |
| Phosphorous | 7.20 | 5.20 | 4.60 | 0.109 |
| Thiamin | 27.00 | 23.80 | 32.10 | 0.003 |
| Riboflavin | 34.90 | 20.10 | 25.90 | <0.0001 |
| Niacin | 35.30 | 28.00 | 34.80 | 0.006 |
| Pyridoxine | 15.60 | 6.30 | 6.70 | <0.0001 |
| Folate | 89.40 | 83.90 | 87.20 | 0.010 |
| Cobalamin | 42.80 | 26.30 | 26.00 | <0.0001 |
| Vitamin C | 36.80 | 27.10 | 32.20 | 0.001 |
| Vitamin D | 100.00 | 99.90 | 100.00 | 0.371 |

^1^Derived from chi-square test..
